# Endothelial cells modulate immune cell responses during atherosclerosis

**DOI:** 10.1016/j.it.2026.03.001

**Published:** 2026-08

**Authors:** Sanne van Kesteren, Lisa Smeehuijzen, Robert Stevenson, Jeffrey Kroon

**Affiliations:** 1 Department of Experimental Vascular Medicine, Amsterdam UMC, location AMC, Meibergdreef 9, Amsterdam, The Netherlands; 2 Amsterdam Cardiovascular Sciences, Atherosclerosis & Aortic Diseases, Amsterdam UMC, Amsterdam, The Netherlands; 3 Laboratory of Angiogenesis and Vascular Metabolism, VIB-KU Leuven Centre for Cancer Biology, VIB, Leuven 3000, Belgium; 4 Laboratory of Angiogenesis and Vascular Metabolism, Department of Oncology, KU Leuven and Leuven Cancer Institute (LKI), Leuven 3000, Belgium

**Keywords:** endothelial cells, atherosclerosis, immune cells, heterogeneity, inflammation, immunomodulation

## Abstract

Endothelial cells (ECs), which form the monolayer lining the vasculature, serve as critical gatekeepers of vascular homeostasis. They actively coordinate immune cell recruitment, adhesion, migration, and activation. Through tightly controlled expression and secretion of chemokines, cytokines, adhesion molecules, and immunomodulatory proteins, ECs locally orchestrate immune responses. In atherosclerosis, ECs contribute to plaque formation, plaque instability, and local inflammation by facilitating immune cell activation and transmigration into the plaque. However, ECs are highly heterogeneous, with their phenotype and function influenced by vascular localization and disease stage. EC subpopulations are transcriptionally distinct and adopt to either proinflammatory or angiogenic phenotypes and differentially participate in immune modulation. This review discusses recent insights into the immunomodulatory functions of ECs in atherosclerosis and underscores the importance of EC heterogeneity in understanding endothelial–immune cell interactions.

## Highlights

Endothelial cells actively regulate immune cell recruitment and behavior, controlling adhesion, activation or suppression, and transmigration throughout all stages of atherosclerotic plaque development.
Endothelial cells communicate with immune cells via an integrated network of signaling molecules. These include chemokines (e.g., C-C motif chemokine ligand 2 and CXC motif chemokine 12), cytokines (e.g., interleukin-1β and interleukin-6), adhesion molecules (e.g., Vascular Cell Adhesion Molecule-1 and Intercellular Adhesion Molecule-1), extracellular vesicles, and immune-modulatory proteins such as PDL-1.
Endothelial cell heterogeneity is stage- and region-dependent within atherosclerotic plaques: among others, specialized inflammatory and angiogenic endothelial cell phenotypes coordinate immune cell recruitment and activation locally, while endothelial cell-derived cytokines/chemokines and extracellular vesicles may propagate low-grade systemic inflammation.

## Significance

Endothelial cells orchestrate local immune responses within atherosclerotic plaques. Understanding the stage- and region-dependent heterogeneity of endothelial cells and their diverse communication routes with immune cells provide crucial insights into vascular inflammation, plaque progression, and opportunities for precisely targeted endothelial-based therapies.

## Endothelial cells as central modulators of atherosclerosis and local immunity

Endothelial cells (ECs) form a dynamic monolayer at the blood–tissue interface and constantly integrate mechanical and biochemical signals, including exposure to **shear stress** (see Glossary), cytokines, metabolites, and nutrients. These inputs orchestrate vascular function by controlling barrier integrity and vascular tone as well as by regulating the transport of nutrients, signaling molecules, and immune cells into the **intima**, thereby shaping local immune responses. This includes leukocyte activation, positioning, and retention within the intima [1]. In atherosclerosis—a chronic inflammatory disease characterized by subendothelial lipid accumulation, sustained immune cell recruitment, and formation of a **fibrous cap** within large- and medium-sized arteries 2, 3—ECs shift from homeostatic gatekeepers to active drivers of plaque initiation and progression (Box 1) [15].

Box 1 **Development of atherosclerotic plaques**

According to the response-to-injury theory [4], atherosclerosis begins when the endothelium becomes chronically stressed or damaged, particularly at arterial bifurcations and curvatures, exposed to low or oscillatory shear stress 5, 6. This dysfunction favors retention of low-density lipoprotein (LDL) within the intima [2], where they become oxidized (oxLDL) and further activate Endothelial cells (ECs) by increasing the expression of leukocyte adhesion molecules, such as ICAM-1 and VCAM-1, cytokine and chemokine secretion, and increased endothelial permeability [6]. Activated ECs recruit circulating immune cells into the intima, where they differentiate into macrophages that engulf oxLDL, becoming lipid-laden foam cells [7]. vSMCs can also migrate from the media to the intima, internalize oxLDL, and contribute significantly to foam cell formation and extracellular matrix formation [8]. Dendritic cells (DCs) have a multifaceted role in plaque formation. A substantial number of foam cells within plaques are derived from DCs [9]. In addition, the binding of oxLDL to scavenger receptors induces cytokine secretion by DCs. DCs may also capture oxLDL within the plaque and present oxLDL-derived antigens to activate lymphocytes, either within proximity or after migrating to lymphatic tissues [9]. Consequently, CD4^+^ T helper cells and CD8^+^ cytotoxic T lymphocytes infiltrate plaques and produce IFN-γ, thereby promoting inflammation and contributing to plaque progression and instability [10]. B cells sustain the inflammatory tone by antibody secretion and antigen presentation [11]. In more advanced stages of atherosclerosis, the lesion progresses to a fibrous plaque, in which Vascular smooth muscle cells (vSMCs) from the tunica media migrate to the intima in response to growth factors produced by foam cells and ECs, where they proliferate and form a fibrous cap at the luminal side of the lesion [2]. A necrotic core forms in the center of the lesion, consisting of apoptotic cells, cellular debris, (phospho)lipids, cholesterol, and extracellular matrix components [12]. Lesion calcification, rupture, and thrombus formation mark the final stages of atherosclerosis. In addition to monocytes, DCs, T cells, and B cells, other immune cells such as neutrophils, NK cells, and mast cells contribute to the microenvironment that drives chronic inflammation. NK cells may reduce plaque stability and increase inflammation through their cytotoxic activity and release of cytokines. Neutrophils release enzymes and reactive oxygen species, while mast cells release mediators such as histamine and proteases that increase endothelial permeability, promote leukocyte recruitment, and destabilize the plaque 13, 14.

Alt-text: Box 1

**Figure 1:**
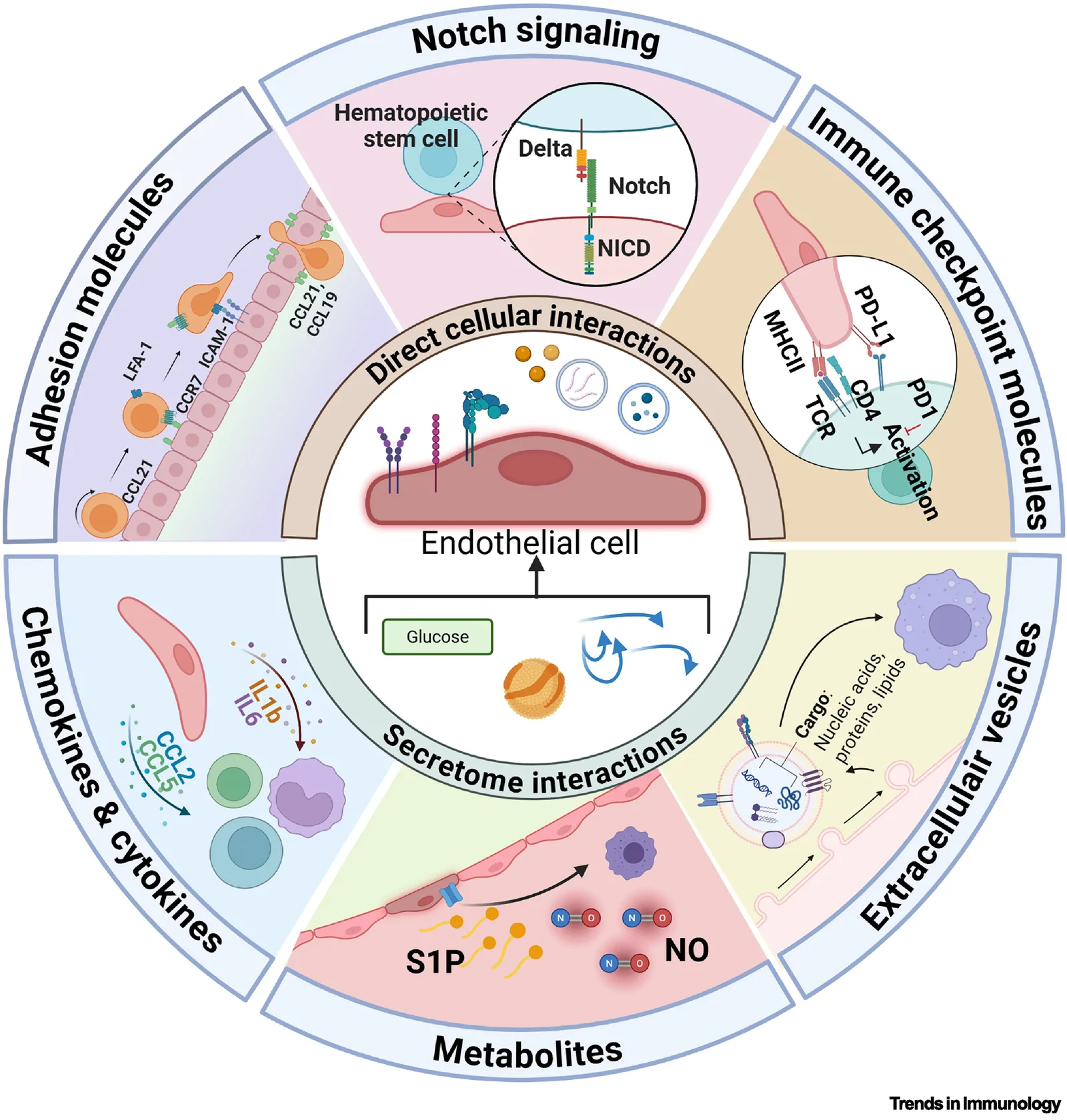
Summary of the endothelial repertoire for crosstalk with immune cells during atherosclerosis. Endothelial cells (ECs) act as dynamic immunomodulators in atherosclerosis, employing a diverse repertoire of molecular mechanisms to communicate with immune cells. Direct interactions include, among others, binding of endothelial adhesion molecules (e.g., VCAM-1 and ICAM-1) to their cognate ligands on immune cells, facilitating leukocyte recruitment and transmigration into the vascular wall. In addition, ECs express immunomodulatory molecules that engage immune cell receptors to elicit proinflammatory or anti-inflammatory responses and shape the inflammatory milieu of the plaque. Beyond direct cell–cell interactions, ECs communicate with immune cells through their secretome. Chemokines and cytokines regulate immune cell recruitment, migration, and activation, while EC-derived metabolites and extracellular vesicles exert additional immunomodulatory effects. Together, these mechanisms position endothelial cells as central orchestrators of immune responses and vascular inflammation in atherosclerosis. Abbreviations: NCID: Notch intracellular domain; MHC: Major Histocompatibility Complex; PD-1: Programmed Death-1; PD-L: Programmed Death-Ligand 1; TCR: T-cell Receptor; S1P: Sphingosine-1-phosphate; NO: Nitric Oxide; IL1β: Interleukin-1beta; IL6: Interleukin-6; CCL2: C-C Motif Chemokine Ligand 2; CCL5: C-C Motif Chemokine Ligand 5; LFA-1: Lymphocyte function-associated antigen-1; VCAM-1: Vascular Cell Adhesion Molecule-1; ICAM-1: ntercellular Adhesion Molecule-1; CCR7: C-C chemokine receptor type 7; CCL21: C-C Motif Chemokine Ligand 21; CCL19: C-C Motif Chemokine Ligand 19. The figure was created using BioRender (https://BioRender.com).

ECs are key regulators of immune responses in the vascular wall. Beyond acting as a physical barrier, they control immune cell recruitment, adhesion, transmigration, activation, and polarization, thereby determining the intensity and quality of inflammation in atherosclerotic plaques. ECs communicate with immune cells through **paracrine** and **endocrine** secretion of chemokines, cytokines, lipids, and other soluble mediators as well as through direct receptor–ligand interactions (including **adhesion molecules** and co-stimulatory/co-inhibitory receptors) (Figure 1) [16]. Through these signaling routes, ECs capture immune cells from the circulation and guide their passage across the endothelial barrier into the plaque, a process known as immune extravasation. This process is mediated by the coordinated expression of endothelial adhesion molecules, which enable sequential leukocyte **tethering**, **rolling**, **firm adhesion**, and **transmigration** (Box 2) [19].

Box 2 **Mechanisms of transendothelial migration of immune cells**

Transendothelial migration (diapedesis) of immune cells into atherosclerotic plaques is tightly regulated by the endothelium and proceeds through three coordinated stages: (1) rolling and tethering, (2) firm adhesion, and (3) transendothelial migration through the endothelial monolayer.

**(1) Rolling and tethering.** Upon endothelial activation at sites of atherosclerotic plaque formation, expression of adhesion molecules such as ICAM-1, VCAM-1, E-selectin (CD62E), P-selectin (CD62P), fibronectin, and PECAM-1 (CD31) is upregulated. In parallel, ECs secrete chemokines such as CCL2 (MCP-1) to recruit monocytes and macrophages via binding to CCR2, and CCL5 (RANTES) to recruit T cells via CCR5. The initial capture of leukocytes from the bloodstream is mediated by selectins that bind their glycoprotein ligands on immune cells. These relatively low-affinity, reversible interactions allow rapid cycles of binding and release, resulting in leukocyte rolling along the endothelial surface 17, 18.

**(2) Firm adhesion.** Rolling leukocytes then encounter chemokines presented on the endothelial surface, which activate leukocyte integrins and increase their affinity for endothelial ligands. This leads to firm adhesion through stronger interactions between endothelial ICAM-1 and VCAM-1 and the leukocyte integrins LFA-1 and VLA-4, respectively. As a result, leukocyte rolling slows and cells arrest on the endothelial surface 19, 20.

**(3) Transendothelial migration.** After arrest, leukocytes crawl over the endothelium to search for permissive exit sites. Before diapedesis, ECs extend ICAM-1- and VCAM-1-rich membrane protrusions that partially surround adherent leukocytes; these so-called ‘transmigratory cups’ are thought to guide leukocytes toward endothelial junctions and facilitate their passage [21]. Leukocytes can then cross the endothelial barrier via paracellular routes, through endothelial junctions, or via transcellular routes, directly through the endothelial cell body, with the paracellular pathway being most common [20]. Diapedesis involves multiple junctional and adhesion proteins, including VE-cadherin, junctional adhesion molecules (JAM-A, JAM-B, and JAM-C), PECAM-1, and CD99 [22]. Under inflammatory conditions such as those present in atherosclerosis, VE-cadherin is phosphorylated and internalized to transiently open junctions, and JAMs are upregulated and redistributed toward the luminal side of endothelial junctions to facilitate leukocyte passage 20, 22.

Alt-text: Box 2

During **immune cell extravasation** and after immune cells have entered the intima, EC-derived signals further shape their activation state, effector functions, and tissue retention. Under physiological conditions, these mechanisms support vascular homeostasis and immune surveillance, whereas in the setting of sustained lipid overload and oxidative stress in atherosclerosis, chronic EC activation promotes exaggerated immune activation and persistent plaque inflammation [23].

Not all ECs behave the same, and this diversity has important consequences for how they shape immune responses and in atherosclerosis 22, 23, 24, 25, 26, 27, 28. Characterization studies of patient-derived carotid plaques and single-cell analysis have revealed pronounced EC heterogeneity, with subtypes differing in gene and protein expression and function [29]. These distinct EC phenotypes likely differ in their capacity to recruit, activate, or even restrain specific immune cell populations during atherosclerosis.

In this review, we discuss recent insights into the repertoire of communication signals that ECs utilize to orchestrate immune modulation during atherosclerosis, and how these signals vary between EC subtypes. These findings thereby hint toward the identification of novel therapeutic targets aimed at modulating vascular inflammation and reducing inflammation-associated comorbidities in atherosclerosis.

## Mechanisms of EC-mediated immune modulation

This section focuses on the molecular and cellular mechanisms through which ECs modulate immune responses during atherosclerosis. We first discuss how hemodynamic forces shape endothelial transcriptional programs, followed by endothelial communication via secreted mediators and **extracellular vesicles** (EVs), and finally direct receptor–ligand interactions at the endothelial surface that regulate leukocyte adhesion and activation.

## Hemodynamic forces shape endothelial immunoregulatory programs

Atherosclerotic plaques develop preferentially at sites of disturbed or low **shear stress**, such as **arterial bifurcations and curvatures**, where **hemodynamic forces** modulate endothelial activation and immune cell recruitment 30, 31. Shear stress directly influences the expression of adhesion molecules, chemokines, and cytokines in ECs, thereby determining the intensity and quality of EC–immune interactions.

### Krüppel-like factor 2: a shear-responsive transcriptional regulator of endothelial quiescence

High **laminar shear stress** is atheroprotective, in part through the induction of the transcription factor Krüppel-like factor 2 (KLF2) in ECs and has been reviewed in detail elsewhere (reviewed in 32, 33, 34). In short, KLF2 maintains endothelial quiescence by promoting nitric oxide production, which preserves the vascular tone [35], and by transcriptionally repressing proinflammatory gene expression in ECs that drive leukocyte recruitment and adhesion, such as Vascular Cell Adhesion Molecule-1 (VCAM-1) and C-C motif chemokine ligand 2 (CCL2) 36, 37. Conversely, low or **oscillatory shear stress** reduces KLF2 expression, thereby promoting inflammation (recently reviewed here [34]).

KLF2 was recently identified as a key factor in the atheroprotective and anti-inflammatory effect of angiopoietin-like 4 (ANGPTL4) in ApoE^-/-^ mice, where ANGPTL4-mediated preservation of KLF2 reduced the expression of CCL2 and VCAM-1 in ECs and reduced monocyte adhesion [38]. KLF2 also promotes the expression of uncoupling protein 2 (UCP2) [39], which attenuates oxidative stress and inflammatory signaling [40].

## The endothelial cell secretome

Hemodynamic forces not only control endothelial gene expression through factors such as KLF2 but also determine the paracrine output of ECs. The endothelial secretome, comprising mediators involved in immune modulation, such as chemokines, cytokines, and EVs, as well as factors that regulate vascular function and tone, including nitric oxide and prostacyclin, collectively coordinates immune responses during atherogenesis.

Among the various components of the endothelial secretome, chemokines and cytokines are key mediators of EC–immune communication, acting through defined receptor–ligand signaling pathways to regulate leukocyte recruitment and activation within the vascular wall.

### Chemokine-mediated leukocyte recruitment

Chemokine production by ECs plays a crucial role in guiding immune cell recruitment to atherosclerotic plaques [41]. For example, CCL2 guides monocytes and macrophages via binding to C-C motif chemokine receptor 2 (CCR2) [24], and C-C motif chemokine ligand 5 (CCL5 or RANTES) attracts T cells via C-C motif chemokine receptor 5 (CCR5) [25]. In a recent *in vitro* work, human aortic ECs (HAECs) were activated with the proatherogenic stimuli IL-1β or calciprotein particles, showing that CCL2, C-C chemokine ligand 7 (CCL7), C-C chemokine ligand 20 (CCL20), and CXC chemokine ligand 8 (CXCL8)—all chemokines involved in immune cell recruitment—are among the highest secreted proteins by activated ECs 42, 43. Chemokines have long been recognized for their capacity to recruit monocytes to sites of atherogenesis, as they are the precursors of macrophages and are originally considered the dominant immune cell type in atherosclerotic lesions [44]. However, more recent studies highlight that T cells are also abundant and functionally important within atherosclerotic lesions [45]. In human carotid plaques, a CXC chemokine ligand 4 (CXCR4)– CXC chemokine ligand 12 (CXCL12) signaling axis between ECs and CD8 T cells was identified, where EC-derived CXCL12 recruited CXCR4-expressing CD8 T cells to **neovessel**-rich regions of the plaque, illustrating how EC chemokine production shapes the spatial organization of adaptive immune cells in atherosclerosis [46].

Beyond this paracrine signaling to immune cells, the CXCR4–CXCL12 axis can also function in an autocrine manner within ECs: EC-derived CXCL12 binding to endothelial CXCR4 promotes endothelial dysfunction and monocyte adhesion that has been positively associated with pulmonary hypertension in mice [47] and was mechanistically demonstrated *in vitro* using monocyte state 1 (MS1) ECs and THP-1 monocytes [48]. However, the role of CXCR4 in atherosclerosis progression is complex: endothelial deletion of CXCR4 worsened plaque formation in ApoE^-/-^ mice [49], whereas increasing endothelial CXCR4 expression by blocking miR-206-3p in ApoE^-/-^ mice reduced monocyte adhesion by decreasing endothelial intracellular adhesion molecule-1 (ICAM-1) expression [50]. Together, these findings illustrate how chemokine signaling governs leukocyte recruitment at atherosclerotic lesions.

### Cytokine-driven amplification of endothelial activation

Cytokines further amplify leukocyte recruitment to atherosclerotic lesions. EC–immune cell communication is bidirectional, as cytokines released by ECs act in autocrine loops to enhance EC activation and leukocyte adhesion, while cytokines produced by infiltrating immune cells feedback on ECs to further reinforce these responses, sustaining immune cell accumulation within plaques. Cytokines, such as tumor necrosis factor alpha (TNFα) and interleukin (IL)-1β, produced by macrophages, T cells, and ECs themselves, are well known to induce ICAM-1 and VCAM-1 expression on ECs via nuclear factor κB (NF-κB) signaling, thereby amplifying immune cell recruitment during atherosclerosis [51]. TNFα, mainly secreted by M1-like macrophages [52] and T helper 1 (Th1) cells [53], increases VCAM-1 surface expression in ECs, and reduction of the TNFα receptor tumor necrosis factor receptor 1 (TNFR1) with brusatol abolishes TNFα-induced ICAM-1 and VCAM-1 expression and THP-1 monocyte adhesion *in vitro* [54]. Similarly, IL-1β, secreted predominantly by ECs upon exposure to oxidized low-density lipoprotein (oxLDL), by monocytes and macrophages, increases ICAM-1 expression in EA.hy926 cells and enhances monocyte adhesion by downregulating miR-1914-5p expression [55]. ECs and immune cells thus participate in self-sustaining loops that drive inflammation and immune cell infiltration into plaques, thereby driving vascular inflammation and atherosclerosis progression.

### Extracellular vesicles as bidirectional mediators of EC–immune crosstalk

While chemokines and cytokines primarily act through receptor-mediated signaling, EVs represent a distinct yet complementary mode of EC–immune crosstalk. EVs are membrane-enclosed vesicles released by virtually all cell types that can both engage in receptor–ligand interactions and deliver bioactive cargo—including proteins, lipids, and RNAs—thereby reprogramming immune and vascular cell behavior in a manner that reflects the activation state of the parent cell. In atherosclerosis, EVs contribute to both proinflammatory and proresolving mechanisms within plaques [56].

### Endothelial extracellular vesicles as activators of leukocytes

Multiple *in vitro* studies have recently demonstrated that ECs secrete EVs that regulate leukocyte adhesion and activation in a shear–stress-dependent manner. Human umbilical vein ECs (HUVECs) cultured under low shear stress (2 dyn/cm^2^) secrete EVs enriched in adhesion molecules and **integrins**, including platelet endothelial cell adhesion molecule (PECAM), melanoma cell adhesion molecule (MCAM), and integrins α5 and β1 [57]. Similarly, EVs derived from disturbed shear–stress-activated HUVECs (4.5 dyn/cm^2^) increase THP-1 monocyte adhesion and upregulate the activation molecules CD80 and CD86 expression in THP-1-derived macrophages, indicating that EC-derived EVs can promote monocyte differentiation into proinflammatory ‘M1-like’ macrophages [58]. Conversely, HUVECs exposed to high laminar shear stress (15 dyn/cm^2^) secrete EVs enriched in miR-34c-4p. When tagged with hyaluronic acid to target CD44^+^ plaque macrophages, these EVs promoted M1-to-M2-like repolarization and enhanced resolution in ApoE^-/-^ mice [59]. These findings suggest that the composition and functional impact of EC-derived EVs are tightly regulated by hemodynamic forces [59].

*Ex vivo* evidence further demonstrates the release of EVs with distinct cargo from their apical versus basolateral surfaces, enabling spatially segregated regulation of immune and stromal cells. More specifically, *ex vivo* IL-1β stimulation of HAECs triggers apical EV release that contained miRNAs involved in T-cell and monocyte activation, while EVs released basolaterally contained miRNAs associated with vascular smooth muscle cell (vSMC) activation and extracellular matrix remodeling [60].

### Leukocyte extracellular vesicles as activators of ECs

Similar to chemokine- and cytokine-mediated signaling, communication between ECs and immune cells via EVs is bidirectional, as EVs released by immune cells can also regulate EC function. In atherosclerosis, LDL oxidation primarily occurs in the vascular wall, yet circulating oxLDL has also been detected in patients 61, 62. *Ex vivo* work with oxLDL-treated primary human monocytes was shown to stimulate the release of proinflammatory cytokines IL-6, IL-1β, and IL-8 [63]. Recent *in vitro* studies show that oxLDL also induces monocytes to release EVs containing miR-146, which, in turn, activate HUVECs to upregulate VCAM-1 and ICAM-1, thereby contributing to leukocyte adhesion [64].

### Intraplaque extracellular vesicles

In addition to circulating monocytes, immune cells residing within plaques also communicate via EVs. Analysis of EV content in carotid plaques and their margins from patients with advanced atherosclerosis revealed that dendritic cell (DC)- and monocyte-derived EVs dominate within plaques, whereas vSMC- and EC-derived EVs predominate at the margins [65]. Moreover, this study predicted that immune-derived EVs in the plaque regulate endothelial function and drive angiogenesis and neovascularization [65]. Together, these findings position EVs as a platform for bidirectional EC–immune crosstalk that shapes the local inflammatory and angiogenic microenvironment in atherosclerotic lesions.

## The endothelial cell interactome

Following chemokine- and cytokine-driven recruitment of immune cells to atherosclerotic lesions, ECs engage infiltrating leukocytes through a diverse repertoire of membrane-bound receptors that directly interact with ligands expressed on immune cells. Through this repertoire of membrane-bound receptors, including adhesion molecules, integrins, and co-stimulatory or co-inhibitory **immune checkpoint proteins**, ECs regulate not only leukocyte adhesion and transmigration but also immune cell activation, polarization, and retention within the vascular wall 23, 66. This EC surface interactome therefore represents a critical layer of immune regulation that complements the secretome to shape the inflammatory microenvironment in atherosclerotic plaques.

### Adhesion molecules orchestrate immune infiltration

The best-characterized examples of adhesion molecules are VCAM-1 and ICAM-1, given their central role in not only early and more advanced stages of atherosclerosis but also other diseases involving endothelial dysfunction, such as cancer, obesity, and kidney diseases [66]. Beyond these canonical adhesion molecules, additional endothelial receptors modulate leukocyte adhesion. Human leukocyte antigens (HLA) class I and Toll-like receptor 4 (TLR4), known for their roles in antigen presentation and pathogen recognition, respectively, can form a signaling complex on HAECs that initiates MyD88-dependent signaling, increases P-selectin surface expression, and enhances monocyte adhesion [67]. Consistently, endothelial dysfunction is positively associated with increased endothelial HLA antigen and adhesion molecule expression in patients with myocardial inflammation, suggesting that such HLA-I-TLR4-driven pathways may also contribute to monocyte adhesion in atherosclerosis [68]. In this way, endothelial adhesion and innate immune receptors act as a tunable gate that determines whether and how leukocytes cross into the plaque.

### Leukocyte-intrinsic control of adhesion

Adhesion and migration of immune cells across the endothelium depend not only on endothelial activation but also on the expression of complimentary receptors and adhesion molecules on immune cells themselves. IL-1α is primarily expressed on circulating monocytes under sterile inflammatory conditions and has recently emerged as a leukocyte-intrinsic regulator of endothelial adhesion [69]. Elevated IL-1α surface expression of on circulating monocytes is positively associated with atherosclerosis in patients with chronic kidney disease and myocardial infraction [69]. *In vitro*, lipopolysaccharide (LPS)-activated peripheral blood mononuclear cells (PBMCs) display increased IL-1α membrane expression which engages IL-1α receptors on HAECs, increasing VCAM-1 expression and monocyte adhesion [69]. This leukocyte-intrinsic regulation of endothelial adhesion suggests that immune cell–derived signals represent actionable targets to modulate leukocyte recruitment in atherosclerosis. In ApoE^-/-^ mice, the anti-inflammatory drug colchicine [70] reduced gene expression of surface adhesion molecules *Selplg* and *Pecam1* in neutrophils and *Sell* in Ly6C^high^ monocytes, thereby inhibiting their recruitment to atherosclerotic lesions [71]. Together, these findings indicate that leukocyte adhesion and migration can be modulated not only by targeting endothelial activation but also by targeting adhesion molecule expression in distinct leukocyte populations.

### Integrin-mediated EC–immune crosstalk under disturbed flow

Among adhesion molecules, integrins constitute a large family of heterodimeric surface receptors expressed by both ECs and immune cells that regulate leukocyte adhesion and EC activation. Beyond transcriptional regulation, integrin activation and clustering at the endothelial surface dynamically modulate EC–immune interactions. Integrin membrane clustering and activation are strongly influenced by hemodynamic forces. In atherosclerosis-prone regions exposed to disturbed or oscillatory flow, aberrant hemodynamic forces promote sustained integrin activation, thereby promoting leukocyte adhesion and activation that contribute to plaque progression [72]. Indeed, blocking disturbed flow-induced integrin α5β1 signaling in ECs protected against endothelial inflammation and plaque formation in ApoE^-/-^ mice [73]. Similarly, oscillatory shear stress activates integrin β3 in ECs, promoting endothelial senescence and inflammation, whereas genetic reduction of *ITGB3* (encoding integrin β3) ameliorated plaque formation in ApoE^-/-^ mice [74]. Hence, integrin expression in ECs forms a substantial mechanism for shaping local immune responses, particularly by regulating leukocyte movement to the site of inflammation [75].

Beyond regulating their own integrin signaling, ECs can also modulate integrin activation on immune cells. For instance, disturbed flow increases endothelial CCRL2 expression in ApoE^-/-^ mice, enabling surface presentation of chemerin that triggers β-integrin activation in monocytes and promotes ICAM-1-dependent adhesion to the endothelium [76]. Together, these findings position integrins as mechanosensitive signaling platforms through which hemodynamic forces shape EC–immune interactions and local inflammatory responses in atherosclerosis.

### Therapeutic targeting of leukocyte–endothelial adhesion

Given the central role of leukocyte adhesion in plaque formation, therapeutic blockade of adhesion pathways is under active investigation. Early strategies focused on reducing endothelial activation and adhesion molecule expression, including VCAM-1, and were shown to effectively decrease leukocyte adhesion *in vitro* (reviewed in [77]). For example, the proteasome inhibitors lactacystin and bortezomib block inhibitor of kappa B (IκB) degradation, thereby preventing NF-κB nuclear translocation and subsequent VCAM-1 transcription; however, animal studies and clinical observations showed severe side effects, including cardiotoxicity and hypertension [77].

More selective approaches make use of antibodies that directly block VCAM-1 binding to α₄-integrin on leukocytes. Recent advances in multicellular culture systems mimicking the atherosclerotic plaque environment demonstrate that antibodies targeting the α₄-integrin-binding site on VCAM-1 are highly effective at reducing monocyte adhesion and transmigration [78]. Notably, antibodies against other VCAM-1 domains may also attenuate monocyte adhesion and migration, albeit to varying extents, because distinct VCAM-1 regions exhibit different affinities for leukocyte ligands and trigger distinct intracellular signaling cascades in ECs [78]. Consequently, domain-specific VCAM-1 blockade may enable the fine-tuning of monocyte recruitment by directly disrupting integrin binding or by modulating endothelial signaling pathways that regulate cell adhesion and migration.

## Endothelial immune checkpoint regulation of leukocyte activation

Beyond controlling leukocyte recruitment and adhesion, ECs also regulate immune cell activation through direct, contact-dependent signaling. In this context, immune checkpoint molecules expressed by ECs deliver co-stimulatory or co-inhibitory signals to leukocytes, thereby locally enhancing or restraining inflammatory responses within atherosclerotic plaques 23, 79. While immune checkpoint blockade has been proven effective in cancer therapy, inhibition of co-inhibitory checkpoints has also been associated with accelerated atherosclerosis, increased plaque size and heightened inflammation in both humans and murine models for atherosclerosis 80, 81.

### PD-L1/PD-1: an endothelial co-inhibitory axis

In patients with heart failure, cardiac ECs located near macrophage-infiltrated regions have been shown to express the co-inhibitory molecule programmed death-ligand 1 (PD-L1) [82]. PD-L1 engages programmed death-1 (PD-1) on T cells and suppresses T-cell activation. In Ldlr^-/-^ mice on a high-fat diet, PD-1 stimulation reduced splenic proinflammatory interferon gamma (IFNγ)–secreting CD4^+^ T cells and cytotoxic CD8^+^ T cells while increasing anti-inflammatory IL-10-secreting CD4^+^ T cells and decreased plaque size [83]. Conversely, blockade of either endothelial PD-L1 or T-cell PD-1 in ApoE^-/-^ mice increased circulating proinflammatory cytokines (TNFα, IFNγ, and granzyme B), lipid accumulation within plaques, and infiltration of proinflammatory CD8^+^ IFNγ^+^ T cells [84] and monocytes [85]. Together, these data suggest that the endothelial PD-L1/PD-1 axis restrains T-cell activation and may limit atherosclerotic inflammation. Similarly, other immune-suppressive proteins such as endothelial C1q exist, which mediate T-cell suppression and numbers in the plaque [86]. However, these findings are based predominantly on murine models, and clinical validation is currently lacking.

### CD40/CD40L: a co-stimulatory pathway driving inflammation

In contrast to the inhibitory PD-1/PD-L1 axis, the co-stimulatory CD40–CD40L pathway promotes vascular inflammation. CD40 is expressed by ECs and is upregulated in response to disturbed shear stress. Circulating T cells and monocytes express the CD40 ligand (CD40L, also known as CD154), and CD40L engagement directly promotes immune cell recruitment and infiltration *in vitro* [87] and in mice [88]. Consistent with a proinflammatory role, myeloid-specific deletion of CD40 in ApoE^-/-^ mice increased the proportion of M2 macrophages in the aorta, indicating that CD40 signaling normally sustains a more inflammatory macrophage phenotype.

Endothelial immune checkpoints thus act as rheostats of the plaque microenvironment, directing it toward inflammation via co-stimulatory pathways such as CD40/CD40L or toward resolution via co-inhibitory axes such as PD-L1/PD-1.

## Endothelial heterogeneity and specialized immune-regulatory subsets in atherosclerotic plaques

Healthy arterial vessels contain two main types of ECs: luminal ECs lining the vessel wall, and **vasa vasorum** ECs in the **adventitia** and intima that supply the arterial wall with nutrients and oxygen [89]. In atherosclerosis, EC heterogeneity increases markedly [90]. During early plaque development, luminal ECs lining the fibrous cap are among the first to respond to inflammatory cytokines and metabolic disturbances. Analyses of human advanced carotid plaques with thin fibrous caps and ruptures showed that neovascularization driven by vasa vasorum ECs becomes increasingly prominent, particularly in plaque **shoulder regions**, where neovessels extend toward the necrotic core and exhibit incomplete endothelial junctions that promote vascular leakage and sustained inflammation [91]. Importantly, immune-related functions differ substantially between luminal and vasa vasorum-derived ECs. ECs in large- and medium-sized luminal arteries are predominantly shaped by mechanosensitive and inflammatory programs, whereas vasa vasorum-derived ECs within plaques are driven primarily by angiogenic programs. Historically, most studies focused on macrovascular ECs; however, neovessel ECs are increasingly recognized as key immunomodulatory players in advanced plaques and are positively correlated with macrophage content 92, 93. In parallel, emerging single-cell ribonucleic acid sequencing (scRNA-seq) and spatial transcriptomic approaches now provide the resolution needed to dissect EC subtype identity and functional specialization^90^, ^94^.

### Transcriptionally distinct endothelial cell subsets in plaques

**Figure 2:**
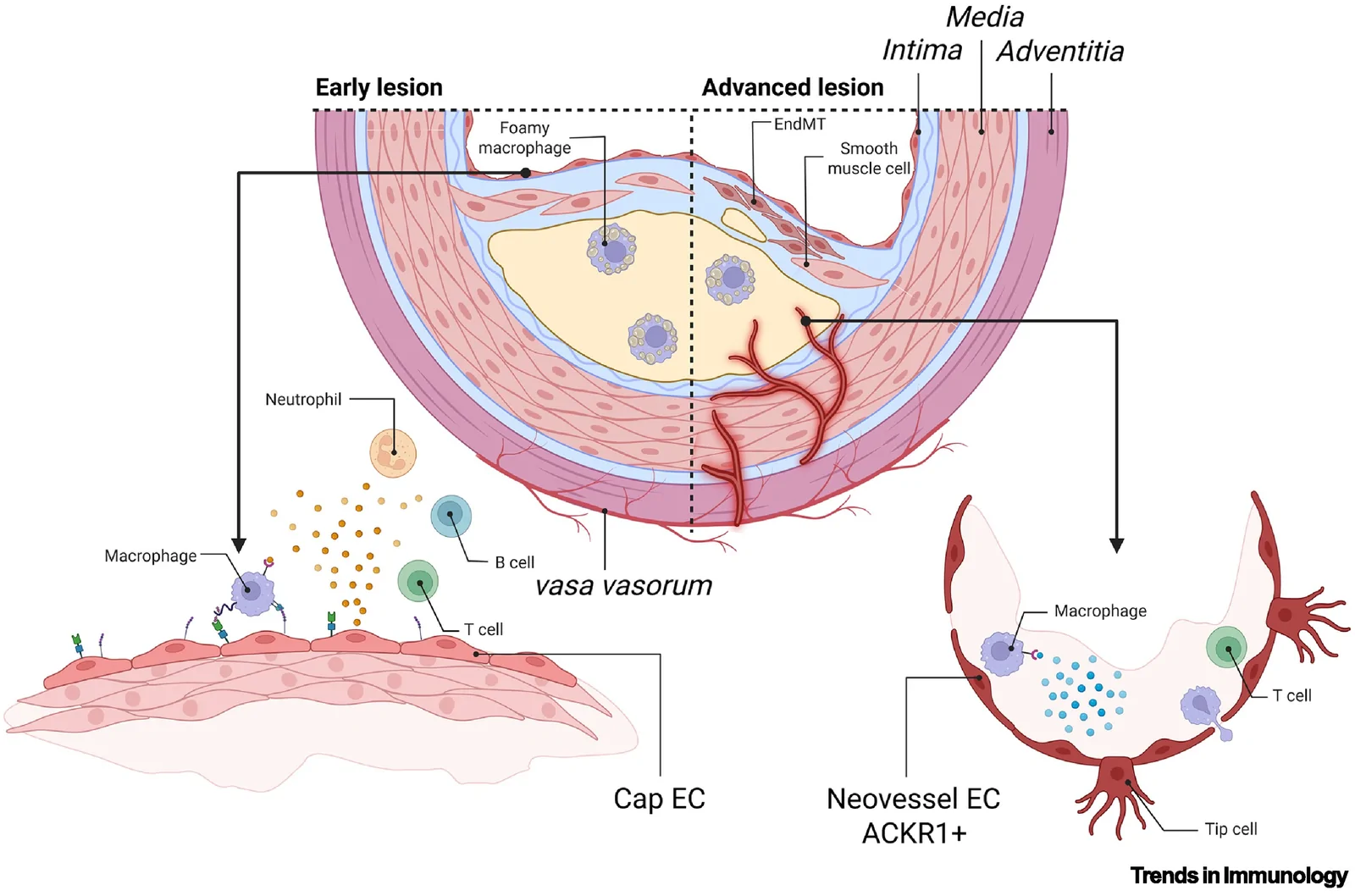
Endothelial heterogeneity specialized in immune cell crosstalk within the atherosclerotic plaque. Early lesion (left): Classical proinflammatory endothelial cells (ECs) are mainly located at the luminal cap of the plaque. These ECs are characterized by high expression of adhesion molecules (ICAM-1, VCAM-1, and SELE) and proinflammatory cytokines and chemokines (CCL2 and interleukin-6), facilitating leukocyte recruitment, activation, and transmigration. They are thought to be more abundant in early lesion development. Advanced lesion (right): Immune-recruiting angiogenic ECs are primarily localized in the adventitia and shoulder regions of advanced plaques. These ECs express ACKR1, microvascular markers (VWF and PLVAP), and adhesion molecules (SELE) and show increased MHC-II machinery (HLA-DRA/B, CD74). They contribute to the formation of leaky neovessels from the vasa vasorum, creating routes for immune cell infiltration, particularly T cells, monocytes, or macrophages. The figure was created using BioRender (https://BioRender.com). EndMT: endothelial-to-mesenchymal transition; CCL2: C-C motif chemokine ligand 2; VWF: Von Willebrand-factor; PLVAP: Plasmalemma vesicle-associated protein; SELE: E-selectine; MHC-II: Major Histocompatibility Complex 2; HLA-DRA: Human Leukocyte Antigen - DR Alpha chain; HLA-DRB: Human Leukocyte Antigen - DR Beta chain; CD74: Cluster of Differentiation 74.

Single-cell transcriptomic profiling of human atherosclerotic plaques has revealed functionally specialized EC subsets defined by distinct gene expression signatures. Rather than reflecting broad activation states, bona fide EC subsets display transcriptional programs specialized for immunomodulation, vascular remodeling, proliferation, or transdifferentiation into vascular smooth musclelike cells via **endothelial-to-mesenchymal transition** (EndMT). Notably, instead of a single ‘immunomodulatory EC’ cell type, recent single-cell and spatial transcriptomic analyses suggest recurring and functionally interpretable EC phenotypes that are relevant for EC–immune crosstalk 95, 96, 97 (Table 1). These subsets show distinct transcriptional specializations for immune cell interaction, including leukocyte recruitment, antigen presentation, and inflammatory cytokine and chemokine signaling. For each of these subtypes, we hypothesize a distinct functional role in modulating immune interactions (Figure 2) 29, 100.

**Table 1:** Single-cell RNA sequenced transcriptional markers of endothelial heterogeneity involved in immune cell crosstalk from human plaque samples

EC-immune cell interacting subtype name	Markers	Function/location	Interaction	Paper
Proinflammatory	SELE, CCL2	Chemokine/adhesion		[95]
Angiogenic	ACKR1, AQP1, FABP4	Vasa vasorum, angiogenesis		[95]
Endothelial_1	ACKR1, HLA genes	Adventitia, antigen presentation, angiogenesis	ACKR1–CCL5 (macrophage)	[96]
C0: ACKR1+ EC	HLA-DRA, HLA-DRB1, CD74, CIITA, ACKR1	Antigen presentation and leukocyte recruitment		[97]
C1: CXCL12+ EC	CXCL12, MKI67, TOP2A, FOXM1	Proliferation, repair, and immune cell interaction		[97]
EC_1 neovessel	ACKR1, BMPR2, COL18A1, THBS1/SERPINE, Deconvolution-based CXCL2 upregulation	Shoulder region plaque	Neighboring with HMOX1+ macrophages	[98] preprint
EC 2 Venular	ACKR1, NR2F2, PLVAP, CXCL12	Neovascular vessels	CXCL12–CXCR4 interaction with CD8+ T cells	[46]
EC1	CD31, CD34, CD49f, CD112, CD200	Inflamed and activated: leukocyte extravasation and integrin signaling, higher abundance in asymptomatic lesions		[99]

### Leukocyte-adhesive luminal ECs

The first immunomodulatory subtype comprises classical proinflammatory ECs, characterized by their transcriptional profile and proinflammatory gene expression among various datasets. Accordingly, the analysis of multiple complementary integrated EC datasets demonstrated an EC subset with upregulated proinflammatory cytokines and chemokines (*IL6*, *CCL2*, and *CXCL14*), and adhesion molecules (*SELE* and *ICAM1*) [95], consistent with proinflammatory, leukocyte-recruiting phenotype. In another patient-derived plaque scRNA-seq dataset, an EC subtype was found enriched for leukocyte adhesion molecules *CD49F* (Integrin α6), *CD112* (Nectin-2), and *CD200* [99]. Pathway analysis linked this cluster to enhanced leukocyte extravasation and integrin signaling. Notably, this subtype was more abundant in asymptomatic lesions when compared with symptomatic lesions [99]. Consistently, molecules supporting leukocyte transmigration, including *VCAM1* and *ICAM1*, have been identified as key genes in atherosclerotic progression, though overall downregulated in human atherosclerotic coronary arteries compared with healthy vessels [101]. Based on these transcriptional and spatial signatures [100], we hypothesize that during early lesion development these proinflammatory ECs could be dominant in the fibrous **cap region** and may serve a dual functional role: on one hand, maintaining vessel wall integrity through adhesion molecule expression, and on the other hand, promoting leukocyte infiltration that contributes to plaque progression.

### Immune-recruiting angiogenic ECs

Extending beyond canonical inflammatory signatures, a comprehensive analysis of spatial and single-cell RNA profiling of human plaques defined an EC population expressing the vasa vasorum marker *ACKR1* together with microvascular markers *VWF* and *PLVAP* as well as adhesion genes such as *SELE*. In aortic ECs, *ACKR1* expression has been linked to modulation of macrophage migration and proinflammatory polarization via the Atypical Chemokine Receptor Secreted Phosphoprotein 1 (osteopontin) (ACKR1)/NF-κB/SPP1 signaling pathway [102], pointing to a role in local immune regulation. Another transcriptomic dataset found that ACKR1^+^ EC subcluster also upregulates *HLA class-II* genes coding for major histocompatibility complex II (MHC II) indicating potential antigen-presenting interactions with CD4^+^ T cells 28, 96. Independent EC-centric atlases further support the presence and immunomodulatory potential of *ACKR1*^*+*^ ECs, showing enrichment for antigen-presentation genes, such as *HLA-DRA*, *HLA-DRB1*, *CD74*, and *CIITA* [97]. Together, spatial scRNA-seq profiling highlights an ACKR1 + EC subset, characterized by coexpression of microvascular markers such as *VWF* and *PLVAP*, along with *HLA class-II* genes. Transcriptomic analyses localize these cells predominantly to the adventitia [96] and plaque shoulder regions in advanced plaques [98]. A cell localization study reveals region-dependent endothelial barrier alterations, with shoulder regions of advanced plaques exhibiting abundant paracellular openings (~8 μm) and higher densities of transcellular pores (0.6–3 μm), whereas the plaque core shows largely intact junctions [103]. These areas of pronounced **endothelial erosion** and increased permeability are enriched with immune cells, while structurally restored regions display minimal infiltration. These spatial niches are also typically characterized by hypoxic and proinflammatory conditions. We therefore hypothesize that ACKR1^+^ EC as angiogenic cells, which promote the sprouting of leaky neovessels from the vasa vasorum. Through this process, they may create potential entry routes for immune cells towards the necrotic core 104, 105. Collectively, these observations suggest that the increasing functional and structural diversity of ECs in advanced plaques is both a response to local plaque microenvironments and a mechanism that could amplify adverse immune responses.

### Repair-associated immunomodulatory ECs

A third group represents a proliferative and repair-associated EC state. These ECs express *CXCL12* and cell-cycle genes, combined with predicted receptor–ligand interactions to immune cells, including natural killer (NK) T cells, macrophages, and plasma cells (e.g., *APP-CD74* and *CD99*) [97], suggesting a role in organizing local immune niches [46]. These *CXCL12*^+^ ECs additionally upregulate *RUNX3*, *TOP2A*, *ZEB1*, *FOXM1*, *MKI67*, *KLF2*, and *RARG* [97], indicative of a proliferative, reparative, and plastic endothelial state that supports angiogenic remodeling, chemokine secretion, and immune cell interactions within the plaque microenvironment. Based on the combination of proliferative and chemokine-expressing signatures, we propose that these ECs not only participate in vascular repair but also help organize the local immune microenvironment. Specifically, CXCL12 secretion may attract CXCR4^+^ lymphocytes and DCs, thereby creating niches that support immune cell retention and activation. Spatial transcriptomic data suggest that interactions between these ECs and CD4^+^ T cells via the CXCL12–CXCR4 axis occur near the necrotic core of advanced human plaques [106]. The active cell cycle state of these ECs could therefore contribute to angiogenic remodeling, which may sustain local immune cell niches by ensuring local perfusion and nutrient supply.

### EndMT: a potential driver of plaque inflammation and instability

Within the spectrum of functionally specialized EC subsets defined by single-cell transcriptomics, EndMT emerges as a phenotype with a profound association with plaque instability [107]. ECs undergoing EndMT partially lose endothelial markers, including junctional proteins, while gaining migratory, proliferative, and extracellular matrix-producing properties (including fibronectin and collagen I expression) [108]. By losing junctional integrity and acquiring mesenchymal traits, EndMT compromises endothelial barrier function, thereby increasing vascular permeability, inflammatory cell infiltration, and lipid accumulation, which collectively drive plaque progression and instability 109, 110. EndMT can be induced by multiple atherogenic stimuli, including inflammatory cytokines (IL-1β, TNF-α, IFN-γ, and TGFβ) 108., 111, intracellular metabolic reprogramming such as altered mitochondrial calcium signaling [112], and **hemodynamic stress** caused by low or oscillatory shear stress [108].

Single-cell CITE-seq atlases of human carotid plaques show that EndMT-derived cells upregulate procoagulant markers, including *CD62P* (P-selectin), *CD61* (integrin β3), and *CD201* (EPCR) [99], and retain the expression of leukocyte adhesion molecules *VCAM1* and *ICAM1* [113]. Whether EndMT ECs express adhesion molecules at levels comparable to or higher than non-EndMT ECs, and whether such expression persists after migration into the intima, remains unclear. *In vitro*, conditioned medium from EndMT ECs alters macrophage responses to oxLDL, reducing their proliferation, antigen-presenting capacity, and TNFα production while increasing scavenger receptor expression and oxLDL uptake [114], indicating functional crosstalk between EndMT ECs and macrophages. However, the specific immune cell populations that interact with EndMT ECs *in vivo* and the mechanisms governing these interactions remain undefined.

## Concluding remarks

ECs are pivotal regulators of immune function and therefore critical regulators of atherosclerosis-related inflammation and plaque development. Through chemokines, cytokines, and adhesion molecules, ECs orchestrate leukocyte recruitment, transmigration, and effector programming within the plaque. Understanding the spatiotemporally regulated dynamics is essential to decipher how endothelial signaling further shapes local immune composition and inflammatory tone. Furthermore, resolving functional differences among EC subsets can clarify how local endothelial signaling guides plaque development and reveal which EC traits might act as predictive markers of plaque instability.

It is important to note that spatial multiomic studies often provide only partial coverage of endothelial phenotypes, which limits functional annotation of identified EC subtypes and highlights the need for further experimental validation. Single-cell and spatial analyses rely on relative calculations within each dataset, meaning that the detected EC populations depend on which cells are present. As a result, not all endothelial states in atherosclerotic plaques may be captured or resolved, and each dataset may highlight a slightly different spectrum of phenotypes. For example, recent single-cell studies have identified reparative-associated ECs with proliferative transcriptional programs [97]. However, these populations may hypothetically also include progenitor-like ECs, which are similarly associated with plaque repair but are rarely detected outside datasets, except for some specific datasets 115, 116.. This variability in detected EC populations influences annotation and may obscure the full landscape of endothelial transcriptional signatures, cellular origins, lineage relationships, and interactions with immune cells.

Building on the concept of increased endothelial heterogeneity during plaque progression, distinct EC subsets present stage-specific therapeutic opportunities for targeting EC–immune cell crosstalk. In addition, as summarized in Table 2, the type and activation state of ECs, as well as the specific immune cells they could interact with, vary across atherosclerosis-related diseases and vascular beds, highlighting disease- and location-specific EC–immune crosstalk.

**Table 2:** Overview of endothelial activation and immune cell involvement across atherosclerosis-related vascular diseases and beds

Disease name	Vascular outcome	Vascular bed prevalence	Endothelial cell activation	Key player immune cells	Research context
Atherosclerosis (common pathological process)	Chronic inflammation resulting in intimal plaque formation, which leads to impaired blood flow	Large- and medium-sized arteries (systemic), systemic microvasculature affected in vasa vasorum during advanced progression (reviewed in [117])	Predominantly aging, shear stress, and lipid driven EC activation (reviewed in [118]): Upregulation of (soluble) adhesion molecules (VCAM-1, ICAM-1), EndMT [90], cytokines (IL-1β, IL-6) 119, 120, NLRP3 [121] inflammasome activation leading to chronic leukocyte recruitment	Heterogeneous group of immune cells (reviewed in 122, 123.) with mainly foamy monocytes/macrophages, T cells [124], but low count of B cells [125]	Clinical study [126], animal models (reviewed in [127]), *in vitro* models (reviewed in [128]), human scRNA-seq profiling (reviewed in 29, 129), human *ex vivo*^90^, ^130^, murine scRNA-seq profiling [131]
Peripheral artery disease (PAD)	Atherosclerotic stenosis outside the arteries	Aorta abdominalis, iliac, femoral, popliteal arteries	Smoking/diabetes driven (reviewed in [118]): Altered proinflammatory angiogenic ATF3/ATF4 ECs [132] and high vSMC content (reviewed in [118])	Increased *LYVE1*^hi^MHCII^low^ macrophages [132] and more monocyte-derived EV release of PD-L2 with increased TIM-3+ CD4+ T cells producing more IL-10 [133]	Clinical studies [133] (reviewed in [134]), human scRNA-seq [124], murine scRNA-seq [131]
Coronary artery disease (CAD)	Coronary atherosclerosis leading to myocardial ischemia	Epicardial coronary arteries, sometimes coronary microvasculature secondary affected	Lipid/shear stress driven (reviewed in [118]) inflammatory adhesion signaling in coronary ECs with unique upregulation of specific chemokines and receptors (e.g., CCL14 and ACKR1) [135] but low vSMC remodeling (reviewed in [118])	Associated with neutrophil influx [136] and macrophage influx [135] and proinflammatory cytokine CCL4 in circulation [137]	Clinical studies [138], human scRNA-seq [124], murine scRNA-seq [125], *in vitro*[139]
Ischemic stroke	Atherosclerotic carotid stenosis leading to cerebral infarction	Carotid arteries, intracranial arteries (Circle of Willis); cerebral microvessels	Ischemic injury triggers persistent Notch1 activation in systemic ECs [140], inducing proinflammatory adhesion phenotype, which leads to blood–brain barrier (BBB) alterations (reviewed in [141])	Increased infiltration of myeloid, NK T cells [142], and neutrophils [143]	Human scRNA-seq of plasma [144], murine bulk RNA-seq 142, 145
Abdominal/inflammatory aortic aneurysm (AAA/IAA)	Arterial dilation with medial wall degradation by intraluminal thrombus	Abdominal aorta or periaortic tissue	Dysregulated EC with Sox18-mediated EndMT [146], disrupted focal junctions, and altered biomechanical sensing 147, 148	Influx of immune cells [149] including (IRF5+PI3Kγ+) macrophage infiltration [150], high infiltration of T cells (reviewed in [151]), neutrophils, NK cells, and plasmacytoid DCs [152]	Human samples 149, 152, murine scRNA-seq 146, 147
Autoimmune-related vascular inflammation [systemic lupus erythematosus (SLE), rheumatoid arthritis, psoriasis]	Increased risk of atherosclerosis (reviewed in 153, 154)	(Systemic) macro- and microvasculature	ECs show activation via increased selectin expression (VCAM/ICAM), and MCP-1 chemokine secretion (mentioned in [155]), and complement pathways [156] with increased expression of selectins, immunoglobulins an chemokine expression (reviewed in [157]), which could lead to endothelial dysregulation	Increased monocyte activation in SLE and psoriasis 156, 158, and increased expression of overlapping immune cells found in atherosclerosis comprising memory B cells, T follicular helper cells, and γδ T cells [159] cells (reviewed in [159])	Human [159], *in vitro*[156]
Chronic kidney disease (CKD)	Systemic endothelial stress with vascular calcification	Renal arteries, glomerular capillaries, systemic arteries	Systemic uremic toxins and oxidative stress chronically activate ECs (reviewed in [160]), increase inflammation and procalcific signaling (reviewed in [161])	Increased monocyte activation (reviewed in [162]), and macrophage activation (reviewed in [163]). Furthermore, increase of (CD3^+^CD31^+^CXCR4^+^) angiogenic T cells [164]	Human samples [164], murine model [165]
Metabolic-associated atherosclerosis (diabetes type 2, obesity)	Accelerated atherosclerosis and microvascular dysfunction (reviewed in [166])	Coronary, peripheral arteries, microvasculature	Hyperglycemia and metabolic/oxidative stress induce endothelial dysfunction, leading to immunometabolic reprogramming with increased adhesion molecules and proinflammatory cytokines 167, 168	Increased monocyte/macrophage 169, 170, neutrophils 171, 172., and T cells (Th1/Th17) [173]	Clinical data 169, 173 multiple murine models (reviewed in [174]), human scRNA-seq 95, 175, murine scRNA-seq [176], *in vitro*[177]
Acute myocardial infarction (AMI)	Acute blockage of coronary artery causing myocardial ischemia	Coronary arteries or cardiac microvasculature	Increased endothelial dysfunction by oxidative stress [178], transient immunoregulatory EC phenotype emerges post-MI with MHCII upregulation, cytokine secretion (IL-6, IL-12) [179] and different EC subtypes involved in proliferation [180] and EndMT [181]	T-cell activation [179], neutrophil and macrophage infiltration [181]	Human scRNA-seq [182], murine scRNA-seq [179], mouse models (reviewed in [183]), *in vitro*[178]
Infectious vascular disease (COVID-19)	Correlates with persistent endothelial injury and increased risk of thrombotic and ischemic events (reviewed in [184])	Pulmonary, coronary, systemic microvasculature (reviewed in [185])	Induce endothelial NF-κB inflammation [186], barrier dysfunction [187], strong leads to leukocyte adhesion molecule upregulation [188], glycocalyx disruption 189, 190, and complement activation [191]	Increase of foamy macrophages [192], NK cells [193]	Leads from patient data [188] (also reviewed in [190]), *in vitro*[186]
Cancer treatment-induced atherosclerosis by immune therapy	Dysregulation of immune response exacerbates risk on vascular damage [194]	Large- and medium-sized arteries (systemic)	Role of endothelium remains unclear (no data), but strong leads of vascular damage (reviewed in [195])	Immune checkpoint inhibitors stimulate T-cell-driven atheroprogression [196]	Clinical data [196] and murine model [196]

In early atherosclerotic plaque development, reducing leukocyte adhesion on ECs may limit plaque infiltration, consequently reducing the local inflammatory response. Hence, it becomes particularly interesting to identify EC subsets specialized in immune cell adhesion. As discussed in this review, ECs in low shear regions secrete EVs promoting leukocyte recruitment and proinflammatory macrophage activation, whereas EC-derived EVs from high laminar shear regions carry anti-inflammatory miRNAs that favor macrophage M2-like polarization 57, 58. Leveraging this stage- and EC-subset-specific EV biology could provide complementary strategies to modulate plaque inflammation and promote repair.

As plaques progress, endothelial heterogeneity further increases, potentially giving rise to regionally specialized EC phenotypes 90, 103, such as ACKR1^+^ or CXCL12^+^ expressing ECs 46, 97. Modulating ACKR1^+^ ECs could influence leukocyte recruitment and antigen presentation, and targeting CXCL12-expressing ECs may reduce monocyte and CD8 T-cell retention and macrophage polarization. Such interventions may allow precise control of plaque immune composition while preserving essential endothelial and immune functions.

Beyond the plaque, endothelial heterogeneity may extend to distinct endothelial states within distant vascular niches, such as the bone marrow, where ECs regulate hematopoiesis and immune cell output. Emerging evidence suggests that endothelial dysfunction in the bone marrow niche can skew hematopoietic programs toward proinflammatory immune cell production, thereby contributing to systemic inflammation, plaque progression, and overall cardiovascular risk [197]. Taken together, the emerging complexity of endothelial heterogeneity highlights major gaps in our understanding of how specific endothelial states regulate immune responses during atherosclerosis. These unresolved issues provide the rationale for several scientific questions (see Outstanding questions).

Mechanistic insights into EC-mediated immune modulation are heavily derived from murine studies, as human samples remain scarce due to the complexity and limited accessibility of atherosclerotic tissue. The spatial multiomic approaches as discussed in this review have enabled phenotypic characterization of EC subsets in human plaques. While this is an important advance, functional validation is still lacking. Additional future studies could integrate spatial profiling with *ex vivo* functional assays on viable human tissue or complex *ex vivo* culture systems [90] to directly assess EC subtype–specific functions and their impact on plaque development.

To conclude, the endothelial secretome and interactome emerge as central regulators of immune cell function in atherosclerosis, with distinct EC subtypes likely differentially engage in leukocyte recruitment, activation, and effector function. These observations highlight the role of ECs as active immune-modulatory cells within vessels and the plaque. While therapeutic modulation of EC–immune interactions represent an attractive future avenue, the primary challenge lies in disentangling the spatial, temporal, and phenotypic complexity of EC–immune crosstalk in cardiovascular disease.

Outstanding questions Which spatially and temporally restricted endothelial subtypes within atherosclerotic plaques are the dominant organizers of local immune niches, and how do they differentially interact with macrophages, T cells, B cells, and innate lymphoid cells?

To what extent does endothelial-to-mesenchymal transition generate endothelial-derived cells that still engage in immune modulation, and how do these endothelial-to-mesenchymal transition-derived cells functionally interact with macrophages and lymphocytes within the plaque?

How do endothelial extracellular vesicles, released apically versus basolaterally, differentially influence circulating immune cells, plaque-resident leukocytes, and vascular smooth muscle cells across distinct plaque regions and disease stages?

Can selective targeting of specific endothelial communication routes (e.g., chemokine axes such as C-C chemokine ligand 2/C-C chemokine receptor 2 (CCL2/CCR2) or CXC motif chemokine ligand 12/CXC motif chemokine ligand 4 (CXCL12/CXCR4), or immune checkpoint pathways such as programmed death-1/programmed death-ligand 1 (PD-1/PD-L1) or cluster of differentiation 40/cluster of differentiation ligand 40 (CD40/CD40L) reprogram plaque immune composition toward a more resolving state without impairing essential endothelial barrier and repair functions?

How can insights from single-cell and spatial profiling of inflammatory, angiogenic, and repair-associated endothelial subtypes be leveraged to reprogram endothelial function, thereby enhancing barrier integrity and reshaping endothelial–immune crosstalk in order to slow or reverse atherosclerotic plaque progression?

How do endothelial signals at the level of the bone marrow niche, which fuel myelopoiesis and systemic inflammation, shape endothelial–immune crosstalk in the plaque, and can coordinated targeting of both compartments more effectively limit atherosclerotic plaque progression than plaque-restricted interventions alone?

Alt-text: Outstanding questions

## Glossary

**Adhesion molecules**: cell-surface proteins that mediate the binding between cells or between cells and the extracellular matrix and are crucial for immune cell recruitment and endothelial interactions (e.g., selectins, integrins, ICAMs, and VCAMs).
**Adventitia**: the outermost layer of a blood vessel, composed mainly of connective tissue, fibroblasts, nerves, and the vasa vasorum; it provides structural support and elasticity.
**Arterial bifurcations and curvatures**: regions where arteries branch or curve, leading to disturbed blood flow patterns and low or oscillatory shear stress, and are therefore prone to atherosclerotic plaque formation.
**Cap region (fibrous cap region)**: the area of an atherosclerotic plaque covered by the fibrous cap, which separates the lipid-rich necrotic core from the blood and is critical for plaque stability.
**Endothelial erosion**: loss or denudation of the endothelial layer overlying a plaque, without rupture of the fibrous cap, exposing the subendothelial matrix and promoting thrombosis.
**Endothelial-to-mesenchymal transition (EndMT)**: a biological process in which endothelial cells lose canonical endothelial markers and functions and acquire mesenchymal-like properties, including increased migration, proliferation, and extracellular matrix production; it is implicated in vascular remodeling and atherosclerosis.
**Extracellular vesicles**: membrane-bound particles released by cells that carry proteins, lipids, and nucleic acids; they mediate intercellular communication and can modulate several cellular processes.
**Fibrous cap**: a layer of connective tissue, composed of mainly collagen and smooth muscle cells that covers the (lipid-rich) core of atherosclerotic plaque and provides structural stability.
**Firm adhesion**: a step in immune cell recruitment, where leukocytes form strong, stable attachments to endothelial cells via integrins, following rolling and activation, prior to transendothelial migration.
**Hemodynamic forces/stress**: the physical forces generated by blood flow and pressure acting on the vessel wall, including shear stress and circumferential stretch, which influence endothelial phenotype and vascular remodeling.
**Immune cell extravasation**: the multistep process by which circulating immune cells exit the bloodstream and migrate across the endothelial barrier into tissues, typically during inflammation.
**Immune checkpoint proteins**: regulatory molecules expressed on immune cells and antigen-presenting cells (e.g., PD-1, CTLA-4, and PD-L1) that modulate immune activation and tolerance, and thereby influence inflammation within atherosclerotic plaques.
**Integrins**: a family of heterodimeric transmembrane receptors that mediate cell–cell and cell–matrix adhesion; they are critical for leukocyte adhesion, migration, and signaling during inflammation.
**Intima**: the innermost layer of a blood vessel, consisting of endothelial cells and a thin subendothelial layer of connective tissue; the primary site of atherosclerotic lesion development.
**Laminar shear stress**: a steady, unidirectional component of flow of blood that exerts uniform frictional force on endothelial cells and generally promotes vascular homeostasis and anti-inflammatory signaling.
**Neovessel**: a small, fragile microvessel that develops within the vessel or atherosclerotic plaque, often originating from the vasa vasorum and associated with intraplaque hemorrhage and inflammation.
**Oscillatory shear stress**: a disturbed flow pattern in which the direction and/or magnitude of shear stress changes throughout the cardiac cycle, typically occurring at bifurcations and curvatures, thereby promoting endothelial dysfunction.
**Rolling**: a stage in the immune cell recruitment cascade during which leukocytes move along the endothelial surface in a slow, intermittent ‘rolling’ fashion, mediated by selectin–ligand interactions.
**Shear stress**: the tangential frictional force per unit area exerted by flowing blood on the endothelial surface; its magnitude and direction shape vascular biology and atherosclerosis.
**Shoulder region**: the area of an atherosclerotic plaque where the fibrous cap meets the adjacent, relatively normal arterial wall; a region of high mechanical stress and a frequent site of plaque rupture.
**Tethering**: the initial, transient attachment of circulating leukocytes to the endothelial surface, mainly mediated by selectins, that precedes rolling and firm adhesion.
**Transmigration**: the process by which leukocytes move across the endothelial layer and basement membrane into underlying tissues, following firm adhesion and activation. A process also referred to as diapedesis or transendothelial migration.
**Vasa vasorum**: small blood vessels that supply oxygen and nutrients to the walls of larger arteries and veins, particularly the adventitia and outer media, and can extend into atherosclerotic plaques.
